# Evaluation of adipokines concentrations in plasma, peritoneal, and endometrioma fluids in women operated on for ovarian endometriosis

**DOI:** 10.3389/fendo.2023.1218980

**Published:** 2023-11-21

**Authors:** Mariusz Wójtowicz, Dariusz Zdun, Aleksander Jerzy Owczarek, Violetta Skrzypulec-Plinta, Magdalena Olszanecka-Glinianowicz

**Affiliations:** ^1^ Clinical Department of Gynecology and Obstetrics, Faculty of Medical Sciences in Zabrze, Medical University of Silesia in Katowice, Katowice, Poland; ^2^ Health Promotion and Obesity Management Unit, Department of Pathophysiology, Faculty of Medical Sciences, Medical University of Silesia, Katowice, Poland; ^3^ Health Promotion and Obesity Management Unit, Department of Pathophysiology, Faculty of Medical Sciences in Katowice, Medical University of Silesia in Katowice, Katowice, Poland; ^4^ Reproductive Health and Sexology Unit, Department of Women’s Health School of Health Sciences in Katowice, Medical University of Silesia in Katowice, Katowice, Poland

**Keywords:** adipokines, nutritional status, endometriosis, inflammation, ovarian endometriosis

## Abstract

**Introduction:**

Some studies indicate the role of selected adipokines in the development of endometriosis. However, a comprehensive assessment of plasma, peritoneal, and endometrioma fluids adipokines concentrations in women with ovarian endometriosis has not yet been performed. Therefore, this study aimed to analyze plasma, peritoneal, and endometrioma fluids selected adipokines concentrations in women operated on for ovarian endometriosis.

**Materials and methods:**

A cross-sectional cohort study involved 56 women operated on for ovarian endometriosis. Body mass, height, and waist circumference were measured, and BMI was calculated. Plasma, peritoneal, and endometrioma fluids adiponectin, leptin, omentin resistin, RBP4, and visfatin/NAMPT were determined by ELISA.

**Results:**

The highest plasma levels of adiponectin, leptin, omentin, and RBP4 than in the endometrioma and peritoneal fluids were found, while levels of resistin and visfatin/NAMPT were significantly higher in endometrioma fluid than in plasma and peritoneal fluid. In addition, levels of visfatin/NAMPT were significantly higher in peritoneal fluid than in plasma. There were also positive correlations between leptin, RBP4, and adiponectin levels in endometrioma and peritoneal fluids (ρ = 0.28; *p* < 0.05; ρ = 0.31; *p* < 0.05; ρ= 0.32; *p* < 0.05, respectively). There were no associations between adipokines levels in plasma, endometrioma, and peritoneal fluids and endometriosis stage.

**Conclusion:**

Our results show that visfatin/NAMPT and resistin may be locally secreted in endometrioma related to inflammation regardless of the stage of endometriosis.

## Introduction

Endometriosis occurs in 2%–10% of women of reproductive age (1). The pathogenesis of endometriosis development is still unclear. The mechanism of pathogenesis of endometriosis is thought to be uterine tissue damage or scarring, the uterine microenvironment, stem cells, remnant cells from menstrual blood, hormones, gene products regulating inflammation, apoptosis, invasion, angiogenesis, autophagy, and oxidative stress (2–4). Macrophages, natural killer cells, T cells, and dendritic cells regulated by cytokines, prostaglandins, and chemokines participate in the initiation and adhesion of endometriosis as well as infertility and pain related to endometriosis (2, 5, 6). Excessive estrogens production play a role in inflammation development (6, 7).

Interestingly, although both obesity and endometriosis are associated with inflammation, the prevalence of endometriosis is inversely related to BMI. However, it has also been shown that abdominal fat distribution is associated with the development of endometriosis (8–10). Some studies showed a role for adipokines in the pathogenesis of endometriosis (8, 11).

The meta-analysis of 25 studies including 2,645 women (1,362 with endometriosis and 1,283 without) showed higher serum leptin levels and leptin/BMI ratio in women with endometriosis. In addition, the leptin levels were lower in women with advanced-stage disease than in women with early endometriosis (12). However, the analysis of data from 29,611 women from the Nurses’ Health Study did not show associations between leptin levels and the development of endometriosis (13). Also, two other meta-analyses did not find differences in plasma leptin levels between women with and without endometriosis, while leptin levels were significantly higher in the peritoneal fluid (14, 15). In addition, higher expression of levels of leptin and leptin-receptor protein was shown in endometrial tissues of women with endometriosis. However, the endometrial leptin mRNA expression was similar in women with and without endometriosis (16). Experimental studies found that leptin enhances the proliferation of both eutrophic and ectopic endometrial stromal cells in endometriosis (17), stimulates the migration and invasion of endometrial cells (18), and was essential for angiogenesis in a mouse model of endometriosis (19). Recombinant adiponectin in levels significantly lower than in serum was found to inhibit the proliferation of primary stromal cells in human endometriosis (20). Moreover, adiponectin reduces the viability of normal endometrial stromal cells (21). However, adiponectin and adiponectin-receptor protein levels in endometrial tissues of women with and without endometriosis were similar (22). Adiponectin levels in the peritoneal fluid were lower in women with than without endometriosis (23) and decreased with the intensity of endometriosis (24). Furthermore, serum adiponectin levels were lower in women with endometriosis and correlated with endometriosis stages (24). However, the meta-analysis of 25 studies mentioned above did not show an association between peritoneal and circulating adiponectin and disease stages but found lower adiponectin levels in women with endometriosis (12). Recently, apelin receptor APLNR was identified as one of three key genes in endometriosis (25).

As was described above, some studies indicated that selected adipokines play a role in the pathogenesis of endometriosis. However, a comprehensive assessment of plasma, peritoneal, and endometrioma fluids adipokines concentrations in women with ovarian endometriosis has not yet been performed. Understanding the relationship between the concentration of adipokines in plasma, peritoneal, and endometrioma fluids can provide data helpful in the diagnosis of ovarian endometriosis. Therefore, this study aimed to analyze plasma, peritoneal, and endometrioma fluids adipokines concentrations in women operated on for ovarian endometriosis.

## Materials and methods

A cross-sectional cohort study involved 56 women operated on for ovarian endometriosis in the Clinical Department of Gynecology and Obstetrics Faculty of Medical Sciences in Zabrze between 2018 and 2022. Inclusion criteria were at least 2 years primary infertility, stage from II to IV ovarian endometriosis, and regular cycles. Endometriosis was diagnosed by laparoscopy and histologically confirmed and classified according to the American Society of Reproductive Medicine classification (26). The exclusion criteria included, other than ovarian localizations of endometriosis, additional extraovarian endometriosis, hormonal disturbances including thyroid dysfunction, Cushing’s syndrome, type 1 and 2 diabetes, smoking and alcohol abuse, changes of body mass during the last 3-month period, and any pharmacological therapy. The study was conducted after obtaining informed consent of each participant, based on the study protocol, approved by the Bioethical Committee of the Medical University of Silesia.

Body mass, height, and waist circumference were measured, and body mass index (BMI) was calculated according to the standard formula. During the morning between 6:00 and 7:00 a.m., after an overnight fast (14 h), 15 mL of venous blood samples were withdrawn. During laparoscopic operation, peritoneal fluid from Douglas’ sinus and endometrioma fluid were collected according to recommendations of the kit manufacturers. Plasma and fluid aliquots were frozen and stored at −70°C.

### Laboratory procedures

Blood morphology and serum C-reactive protein (CRP) levels were assessed. CRP concentrations were assessed by an automated system (Modular PPE, Roche Diagnostics GmbH, Mannheim, Germany). The inter-assay coefficient of variability was 5.7%.

The ELISA method was used for measurements of plasma and fluids leptin (TECOmedical AG Sissach, Switzerland), adiponectin (TECOmedical AG Sissach, Switzerland), omentin (DRG Instruments GmbH, Marburg, Germany), RBP4 levels (Phoenix Pharmaceuticals, Burlingame, USA), resistin (R&D, Minneapolis, MN, USA), and visfatin/NAMPT (BioVendor, Brno, The Czech Republic) with the LoQ of 0.08 ng/mL, 0.11 ng/mL, 0.2 ng/mL, 0.6 ng/mL and 0.5 ng/mL, 2.17 ng/mL, 0.05 ng/mL, and 30 pg/mL respectively; intra- and inter-assay coefficients of variations were 4.6% and 7% for leptin, 5% and 6% for adiponectin, 3.7% and 4.6% for omentin-1, 5.0% and <14.0% for RBP4, <5.5% and <9.2% for resistin, and 5.6% and 5.9% for visfatin/NAMPT.

### Statistical analysis

Statistical analysis was performed using STATISTICA 13.0 PL (TIBCO Software Inc., Palo Alto, CA, U.S.) and StataSE 13.0 (StataCorp LP, TX, U.S.). Statistical significance was set at a *p* < 0.05. All tests were two-tailed. Nominal and ordinal data were expressed as percentages. Interval data were expressed as mean ± standard deviation (normal distribution) or median (lower–upper quartiles). The distribution of variables was evaluated by the *W* Shapiro-Wilk test and the quantile–quantile (Q–Q) plot. Rank ANOVA was used to compare adipokines levels between plasma and endometrial/peritoneal fluids with Tukey as a *post-hoc* test. The homogeneity of variance was assessed by the *F* Fisher–Snedecor test. Correlation between variables was assessed with the ρ Spearman rank correlation coefficient.

## Results

The baseline characteristics of the study group are presented in **Table 1**.

**Table 1:** Baseline characteristics of the study group.

N	56
Age [years]	33 ± 6
CRP [mg/L]	0.6 (0.6–1.8)
HGB [g/dL]	13.1 ± 1.1
WBC [tys/μL]	6.8 (5.6–8.1)
RBC [mln/μL]	4.4 ± 0.4
PLT [tys/μL]	258 ± 51
BMI [kg/m^2^]	22.6 ± 4.3
Stages of endometriosis [*N*(%)]	
II	11 (26.4)
III	28 (52.8)
IV	14 (26.4)

Mean ± standard deviation or median (lower quartile–upper quartile).

Statistically significant higher plasma levels of adiponectin, leptin, omentin, and RBP4 compared to endometrioma and peritoneal fluids were found. Levels of resistin and visfatin/NAMPT were significantly higher in endometrioma fluid than in plasma and peritoneal fluid. In addition, levels of visfatin/NAMPT were significantly higher in peritoneal fluid than in plasma (**Table 2**).

**Table 2:** Adipokines levels in plasma and endometrioma and peritoneal fluids.

	Plasma	Endometrioma fluid	Peritoneal fluid	*p*
Adiponectin [μg/mL]	9.8 (7.3–13.1)	1.2^#^ (0.1–2.4)	0.1^#^ (0.0–1.3)	<0.001
Leptin [ng/mL]	16.6 (10.1–29.5)	2.2^#^ (1.0–4.4)	1.6^#^ (0.4–4.1)	<0.001
Omentin [mg/mL]	498.6 (388.9–631.9)	228.0^*^ (90.1–702.9)	230.9 (83.0–945.2)	<0.01
Resistin [ng/mL]	5.6 (4.6–6.8)	71.0^#^ (44.7–96.4)	3.9^$^ (1.0–42.6)	<0.001
RBP4 [μg/L]	29.4 (24.0–34.0)	5.5^#^ (2.9–8.7)	1.8^#^ (1.1–3.8)	<0.001
Visfatin/NAMPT [ng/mL]	1.2 (0.4–2.3)	12.0^#^ (8.1–13.4)	7.0^#$^ (1.4–12.1)	<0.001

^*^p < 0.05, ^#^p < 0.001 in comparison to plasma levels; ^$^p < 0.01 in comparison to endometrial fluid; median (lower quartile–upper quartile).

Spearman rank correlation analysis showed a positive correlation between plasma leptin levels and BMI (ρ = 0.63; *p* < 0.00) and negative correlations between plasma omentin and BMI (ρ = −0.25; *p* = 0.07). There were no correlations between plasma levels of other adipokines levels and BMI. In addition, adipokines levels in endometrioma and peritoneal fluids did not correlate with BMI.

The positive correlation between visfatin/NAMPT levels in peritoneal fluids and WBC numbers was found (ρ = 0.31; *p* < 0.05). There were also positive correlations between leptin levels in endometrioma and peritoneal fluids (ρ = 0.28; *p* < 0.05), between leptin levels in endometrioma fluids and WBC numbers (ρ = 0.32; *p* < 0.05), and between plasm leptin levels and serum CRP levels (ρ = 0.33; *p* < 0.05). We observed positive correlations between RBP4 levels in endometrioma and peritoneal fluids (ρ = 0.31; *p* < 0.05) and between RBP4 levels in plasma and endometrioma fluids (ρ = 0.44; *p* < 0.001), as well as RBP4 levels in endometrioma and peritoneal fluids and WBC numbers (ρ = 0.33; *p* < 0.05 and ρ = 0.33; *p* < 0.05, respectively). In addition, there was also positive correlation between plasma and endometrioma fluid RBP4 levels and serum CRP levels (ρ = 0.37; *p* < 0.05 and ρ = 0.29; *p* < 0.05, respectively). A positive correlation between adiponectin levels in endometrioma and peritoneal fluids (ρ = 0.32; *p* < 0.05) was found. Moreover, a negative correlation between plasma omentin levels and serum CRP levels (ρ = −0.40; *p* < 0.01) was observed.

There were no associations between adipokines levels in both plasma and endometrioma and peritoneal fluids and endometriosis stage.

## Discussion

To the best of our knowledge, this is the first study that assessed the levels of adipokines in plasma, endometrioma, and peritoneal fluids of women operated on for ovarian endometriosis. Our study showed significant higher plasma levels of adiponectin, leptin, omentin, and RBP4 than in endometrioma and peritoneal fluids. In addition, there were no associations between adiponectin, leptin, omentin, and RBP4 levels in either plasma, endometrioma, or peritoneal fluids and endometriosis stage. Our results contradict previous studies that showed significantly higher leptin levels in peritoneal fluid than in plasma of women with endometriosis (14, 15) and higher levels of leptin in endometrial tissues (16). Moreover, contradictory to a study that showed a decrease of adiponectin levels in peritoneal fluid with the intensity of endometriosis (24), we did not observe any associations between adiponectin levels in either plasma, endometrioma, or peritoneal fluids and endometriosis stage. However, similar to a meta-analysis of 25 studies (12), we did not observe associations between plasma and peritoneal fluid adiponectin levels and disease stage. It should be noted that our study showed positive correlations between adiponectin, leptin, and RBP4 levels in endometrioma and peritoneal fluids. In addition, only plasma RBP4 levels correlated with RBP4 levels in endometrioma fluid. Moreover, the positive correlation between RBP4 levels in both endometrioma and peritoneal fluids and WBC numbers and between RBP4 levels in both plasma and endometrioma fluid and serum CRP levels was observed. So far, only one study showed higher RBP4 levels in peritoneal fluid in the women with than without endometriosis. Moreover, RBP4 immunoreactivity was significantly higher in ovarian endometriomas of women with advanced-stage endometriosis compared to women without endometriosis. Furthermore, *in vitro*, human recombinant-RBP4 increased the invasiveness of endometrial stromal cells. Transfection with RBP4 siRNA reduced the viability and invasiveness of endometrial stromal cells (27). Further studies are necessary to clarify the role of RBP4 in the pathogenesis of endometriosis. Although our study suggests that RBP4 levels in both endometrioma and peritoneal fluids reflect the severity of inflammation in endometriosis, it also seems that leptin levels in endometrioma fluid are an inflammation marker because our study has shown correlation between its levels in endometrioma fluid and WBC number.

Of interest, our study found significantly higher levels of resistin and visfatin/NAMPT in endometrioma fluid than in plasma and peritoneal fluid. Moreover, levels of visfatin/NAMPT were significantly higher in peritoneal fluid than in plasma. Furthermore, a positive correlation between visfatin/NAMPT levels in peritoneal fluids and WBC numbers was found. Visfatin is an adipokine mainly expressed and secreted by macrophages and adipocytes of visceral adipose tissue, and is also known as nicotinamide phosphoribosyltransferase (NAMPT) and pre-B-cell colony enhancing factor (PCEF) produced by lymphocytes (28–30). Thus, the higher visfatin/NAMPT levels in endometrioma and peritoneal fluids and their correlation with WBC numbers may indicate that it is an indicator of inflammation associated with endometriosis. Our results are contrary to a study that has shown lower visfatin levels in peritoneal fluid in women with than without endometriosis (31). However, the results of another study that found significantly lower visfatin gene expression in whole blood samples in women with than without endometriosis (32) seem to support our hypothesis that increased visfatin concentrations in endometrioma and peritoneal fluids are associated with its local production in endometrioma cells. Resistin is the adipokine produced by macrophages of visceral adipose tissue. However, the primary sources of circulating resistin in humans are peripheral blood mononuclear cells (PBMCs), macrophages, and bone marrow cells (33). Thus, we hypothesized that higher resistin levels in endometrioma fluid is similar to visfatin/NAMPT is an indicator of local inflammation in endometriosis. Our hypothesis is supported by studies that showed higher resistin levels in peritoneal fluid of women with than without endometriosis (34) and higher resistin mRNA and protein levels in ectopic endometrial tissue of patients with endometriosis compared to normal eutopic endometrial tissue (35).

The limitations of our study are the size of the study group and the lack of a control group without endometriosis. Furthermore, the distribution of body fat and its visceral deposits was not directly assessed. Moreover, in our study, only selected adipokines were analyzed.

The strengths of the study include the demonstration that the analysis of visfatin/NAMPT and resistin concentrations in the peritoneal fluid may be clinically useful in assessing the severity of inflammation in ovarian endometriosis. A pouch of Douglas fluid collection is an easier procedure than an ovarian endometriosis fluid collection. Inflammation can translate into increased pain and fertility disturbances. However, it should be emphasized that our study opens a new direction of research on ovarian endometriosis, and at this stage, it cannot be used as guidelines for clinical management. More research is needed to translate the evidence into clinical utility.

## Conclusions

Our results show that visfatin/NAMPT and resistin may be locally secreted in endometrioma related to inflammation regardless of the stage of endometriosis.

## Data availability statement

The raw data supporting the conclusions of this article will be made available by the authors, without undue reservation.

## Ethics statement

The studies involving human participants were reviewed and approved by Ethics Committee of Medical University of Silesia Katowice Poland. The patients/participants provided their written informed consent to participate in this study.

## Author contributions

MW has initial idea designed the study protocol, performed an operation, supervised the blood and fluids collections, and wrote the manuscript; DZ performed an operation, collected the blood and fluids, collected informed consents from the participants, and search literature, AO performed the statistical analysis and prepared the tables and figures, VS-P has initial idea, received grant, and revised the manuscript; MO-G has an initial idea, supervised the study, performed the data analysis and revised the manuscript. All authors contributed to the article and approved the submitted version.
